# Hsp40 Gene Therapy Exerts Therapeutic Effects on Polyglutamine Disease Mice via a Non-Cell Autonomous Mechanism

**DOI:** 10.1371/journal.pone.0051069

**Published:** 2012-11-30

**Authors:** H. Akiko Popiel, Toshihide Takeuchi, Hiromi Fujita, Kazuhiro Yamamoto, Chiyomi Ito, Hiroshi Yamane, Shin-ichi Muramatsu, Tatsushi Toda, Keiji Wada, Yoshitaka Nagai

**Affiliations:** 1 Department of Degenerative Neurological Diseases, National Institute of Neuroscience, National Center of Neurology and Psychiatry, Kodaira, Tokyo, Japan; 2 Division of Laboratory Animals Resources, National Institute of Neuroscience, National Center of Neurology and Psychiatry, Kodaira, Tokyo, Japan; 3 Division of Neurology/Molecular Brain Science, Kobe University Graduate School of Medicine, Kobe, Hyogo, Japan; 4 Division of Neurology, Department of Medicine, Jichi Medical University, Shimotsuke, Tochigi, Japan; 5 Core Research for Evolutional Science and Technology (CREST), Japan Science and Technology Agency, Kawaguchi, Saitama, Japan; Tokyo Medical and Dental University, Japan

## Abstract

The polyglutamine (polyQ) diseases such as Huntington’s disease (HD), are neurodegenerative diseases caused by proteins with an expanded polyQ stretch, which misfold and aggregate, and eventually accumulate as inclusion bodies within neurons. Molecules that inhibit polyQ protein misfolding/aggregation, such as Polyglutamine Binding Peptide 1 (QBP1) and molecular chaperones, have been shown to exert therapeutic effects *in vivo* by crossing of transgenic animals. Towards developing a therapy using these aggregation inhibitors, we here investigated the effect of viral vector-mediated gene therapy using QBP1 and molecular chaperones on polyQ disease model mice. We found that injection of adeno-associated virus type 5 (AAV5) expressing QBP1 or Hsp40 into the striatum both dramatically suppresses inclusion body formation in the HD mouse R6/2. AAV5-Hsp40 injection also ameliorated the motor impairment and extended the lifespan of R6/2 mice. Unexpectedly, we found even in virus non-infected cells that AAV5-Hsp40 appreciably suppresses inclusion body formation, suggesting a non-cell autonomous therapeutic effect. We further show that Hsp40 inhibits secretion of the polyQ protein from cultured cells, implying that it inhibits the recently suggested cell-cell transmission of the polyQ protein. Our results demonstrate for the first time the therapeutic effect of Hsp40 gene therapy on the neurological phenotypes of polyQ disease mice.

## Introduction

The polyglutamine (polyQ) diseases are a group of inherited neurodegenerative disorders that are all caused by a common genetic mutation, namely an expansion (>40) of a polyQ-encoding CAG repeat in each unrelated disease-causing gene [1], [2]. Nine polyQ diseases have been identified to date, including Huntington’s disease (HD), spinocerebellar ataxia (SCA) types 1, 2, 3, 6, 7, 17, dentatorubral pallidoluysian atrophy (DRPLA), and spinobulbar muscular atrophy (SBMA). In these disorders, progressive degeneration of neurons in brain areas specific for each disorder occurs, causing various neurological and psychiatric symptoms corresponding to each affected brain area [1], [2].

In the common molecular pathogenesis of the polyQ diseases, proteins with an expanded polyQ stretch become misfolded and form aggregates, and subsequently accumulate as inclusion bodies within neurons, eventually resulting in neurodegeneration [3]–[7]. Moreover, recent studies suggest that prion-like transmission of aggregation-prone proteins between cells is involved in neuropathological spreading during disease progression in not only the polyQ diseases but also various other neurodegenerative diseases [8]–[11]. Although various therapeutic strategies against downstream targets of the pathogenic cascade have been investigated, misfolding and aggregation of the polyQ protein are ideal therapeutic targets since they are the most initial pathogenic events, and therefore their inhibition is expected to result in the suppression of a broad range of downstream pathogenic events [3], [5], [12], [13].

In our attempt to establish a therapy for the polyQ diseases, we hypothesized that molecules that specifically bind to the expanded polyQ stretch would suppress misfolding and aggregation of the expanded polyQ protein. Accordingly, by phage display screening of combinatorial peptide libraries, we identified PolyQ Binding Peptide 1 (QBP1), and proved that QBP1 indeed inhibits polyQ protein misfolding/aggregation *in vitro* [14]–[16]. Furthermore, we demonstrated that expression of QBP1 suppresses neurodegeneration *in vivo* in polyQ disease model animals [16], [17]. Another approach for targeting misfolding and aggregation of the expanded polyQ protein is to utilize molecular chaperones, which are a group of biomolecules that assist the proper folding of proteins and prevent protein misfolding/aggregation [18]–[20]. Indeed, overexpression of molecular chaperones such as Hsp40 and Hsp70 has been shown to suppress polyQ protein aggregation and polyQ-induced neurodegeneration in various animal models of the polyQ diseases, such as flies [21]–[24], worms [25] and mice [26]–[30]. However, most studies showing the therapeutic efficacy of these aggregation inhibitors have been performed by crossing transgenic animals so far. To develop a therapy using aggregation inhibitors such as QBP1 and molecular chaperones, transgenes need to be delivered in affected individuals by administration, rather than be expressed in the next generation by crossing transgenic animals.

In this study, we employed a viral vector to deliver these transgenes into the brain and investigated their therapeutic effects on polyQ disease model mice. Among various viral vectors, we chose to use adeno-associated virus vector (AAV) because of its widespread infection throughout the brain, its long-term expression of transgenes, and its safety [31], [32]. We successfully demonstrate the therapeutic effects of AAV5-QBP1 and AAV5-Hsp40 injections on a mouse model of HD. Most interestingly, we found that AAV5-Hsp40 exerts a non-cell autonomous therapeutic effect, possibly via inhibition of the recently-suggested cell-cell transmission of the polyQ protein, indicating a novel therapeutic mode of action of Hsp40.

## Results

### AAV5-QBP1 and AAV5-Hsp40 Inhibit Inclusion Body Formation in polyQ Disease Mouse Neurons

**Figure 1:**
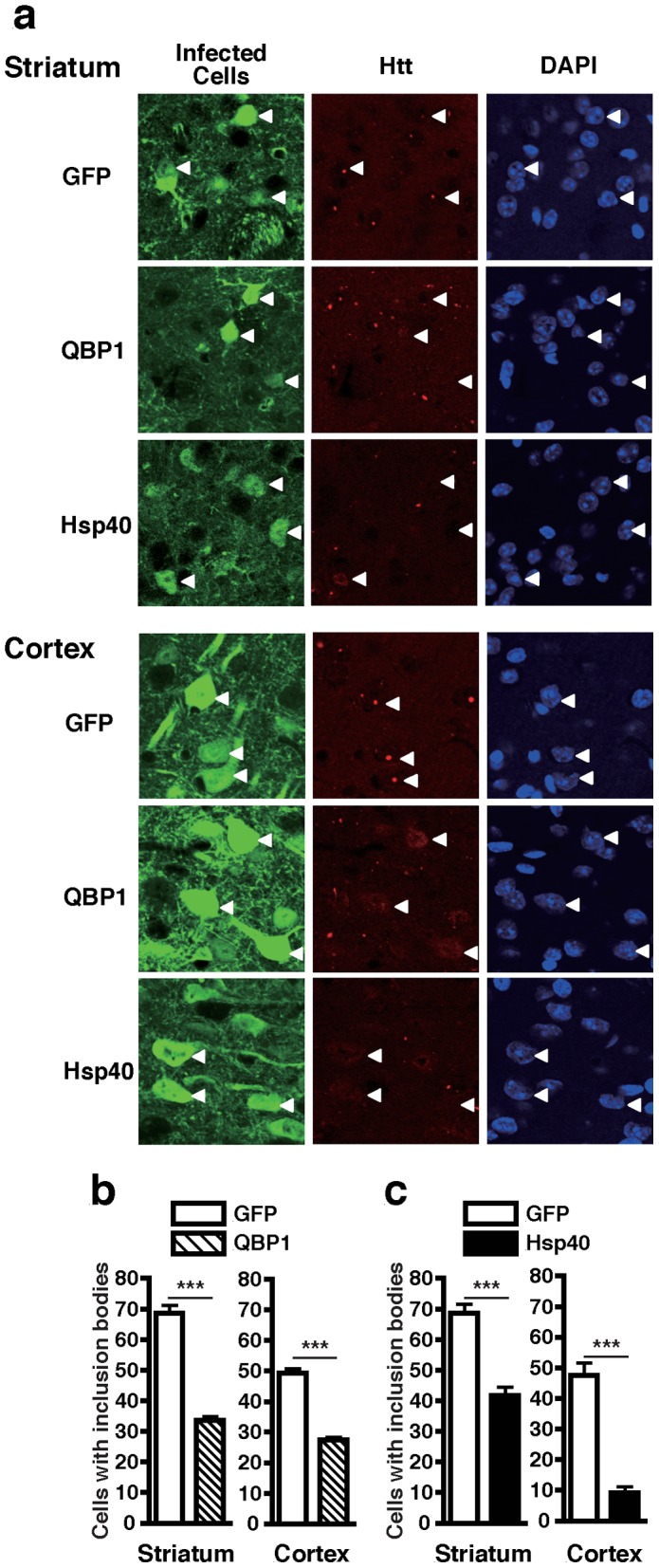
AAV5-QBP1 and AAV5-Hsp40 inhibit polyQ inclusion body formation in virus infected neurons of polyQ disease mice. R6/2 mice at P7 were injected with AAV5-GFP on one side of the striatum and either AAV5-QBP1 or AAV5-Hsp40 on the other side, and htt inclusion body formation in virus infected neurons was assessed at 8 weeks of age by immunohistochemistry of brain sections. (A) Representative photographs of striatal (top panels) and cortical (bottom panels) sections of R6/2 mice, injected with the indicated viruses. Green; virus infected cells, red; htt, and blue; nuclei visualized by DAPI staining. In each panel, representative virus infected cells are indicated by white arrowheads. (B) Inclusion body formation in AAV5-QBP1 infected neurons in the striatum (left) and cortex (right). (C) Inclusion body formation in AAV5-Hsp40 infected neurons in the striatum (left) and cortex (right). In (B) and (C), data are shown as means ± SEM of ≥6 fields of view, in which over 180 cells were counted (*p<0.05, ***p<0.001). Representative results of two mice analyzed are shown.

We employed the R6/2 HD mouse model [33] to investigate the therapeutic effect of AAV-mediated expression of QBP1 and molecular chaperones. We first tested the effect on accumulation of the pathogenic polyQ protein into inclusion bodies in the neurons of R6/2 mice by AAV5 injections. Injections were performed on mice at postnatal day 7 (P7) using an infusion pump (see Materials and Methods), which has been shown to lead to widespread delivery of the injected molecules in the brain [34], and indeed resulted in widespread expression of the transgene throughout the injected brain hemisphere with ∼30% infection efficiencies (Fig. S1). R6/2 mice were injected with AAV5-GFP on one side of the striatum and AAV5-QBP1 on the other side, and htt inclusion body formation was compared between the two sides of both the striatum and the cortex. Inclusion bodies were already formed in 36.0% of AAV5-GFP infected neurons in the striatum at 4 weeks of age, which increased to 68.5% at 8 weeks and 73.8% at 14 weeks, and an age dependent increase in inclusion bodies was also observed in the cortex (Fig. S2). In contrast, AAV5-QBP1 infected neurons had significantly less inclusion bodies at most time points (Fig. S2), and the rates of neurons with inclusion bodies at 8 weeks, for example, were 68.5% for GFP vs 33.7% for QBP1 (*p*<0.001) in the striatum, and 49.3% for GFP vs 27.4% for QBP1 (*p*<0.001) in the cortex (Figs. 1A,B). These results demonstrate a significant inhibitory effect of AAV5-QBP1 on inclusion body formation.

We also tested the effect of AAV5-mediated expression of a molecular chaperone on inclusion body formation. Among the various molecular chaperones, we chose to use a member of the Hsp40 family, namely DNAJB1 (referred to as Hsp40 in this study), since members of the DNAJB subfamily have been reported to be the most potent suppressors of expanded polyQ protein aggregation and toxicity [35]. The effect of AAV5-Hsp40 on polyQ inclusion body formation in R6/2 mice was investigated at 8 weeks, an age at which AAV5-QBP1 showed a clear inhibitory effect. AAV5-Hsp40 also exerted a robust effect on inclusion body formation, and the rates of virus infected neurons with inclusion bodies were 68.6% for GFP vs 41.7% for Hsp40 (*p*<0.001) in the striatum, and 47.5% for GFP vs 9.2% for Hsp40 (*p*<0.001) in the cortex (Figs. 1A,C). This difference in the effectiveness of Hsp40 between the two brain areas may be due to differences in the expression levels of its partner Hsp70. Although whether polyQ inclusion bodies themselves are cytotoxic or cytoprotective has been controversial [36], we assume that suppression of inclusion body formation by QBP1 and Hsp40 can be regarded as a therapeutic effect, since they act by preventing the initial toxic misfolding of the polyQ protein and promoting its refolding, respectively [15], [16], [19], [20]. Therefore, these results collectively demonstrate that QBP1 and Hsp40 exert therapeutic effects in polyQ disease mouse neurons, via their widespread and long-term expression using AAV5.

### AAV5-Hsp40 Improves Neurological Phenotypes of polyQ Disease Mice

Since our above results demonstrated that AAV5-QBP1 and AAV5-Hsp40 inhibit accumulation of the pathogenic polyQ protein into inclusions, we next tested whether this could also lead to amelioration of the neurological phenotypes of R6/2 mice. For this purpose we injected AAV5-GFP, AAV5-QBP1, or AAV5-Hsp40 into both sides of the striatum of P7 R6/2 mice, and analyzed their effects on various neurological phenotypes of these mice.

**Figure 2:**
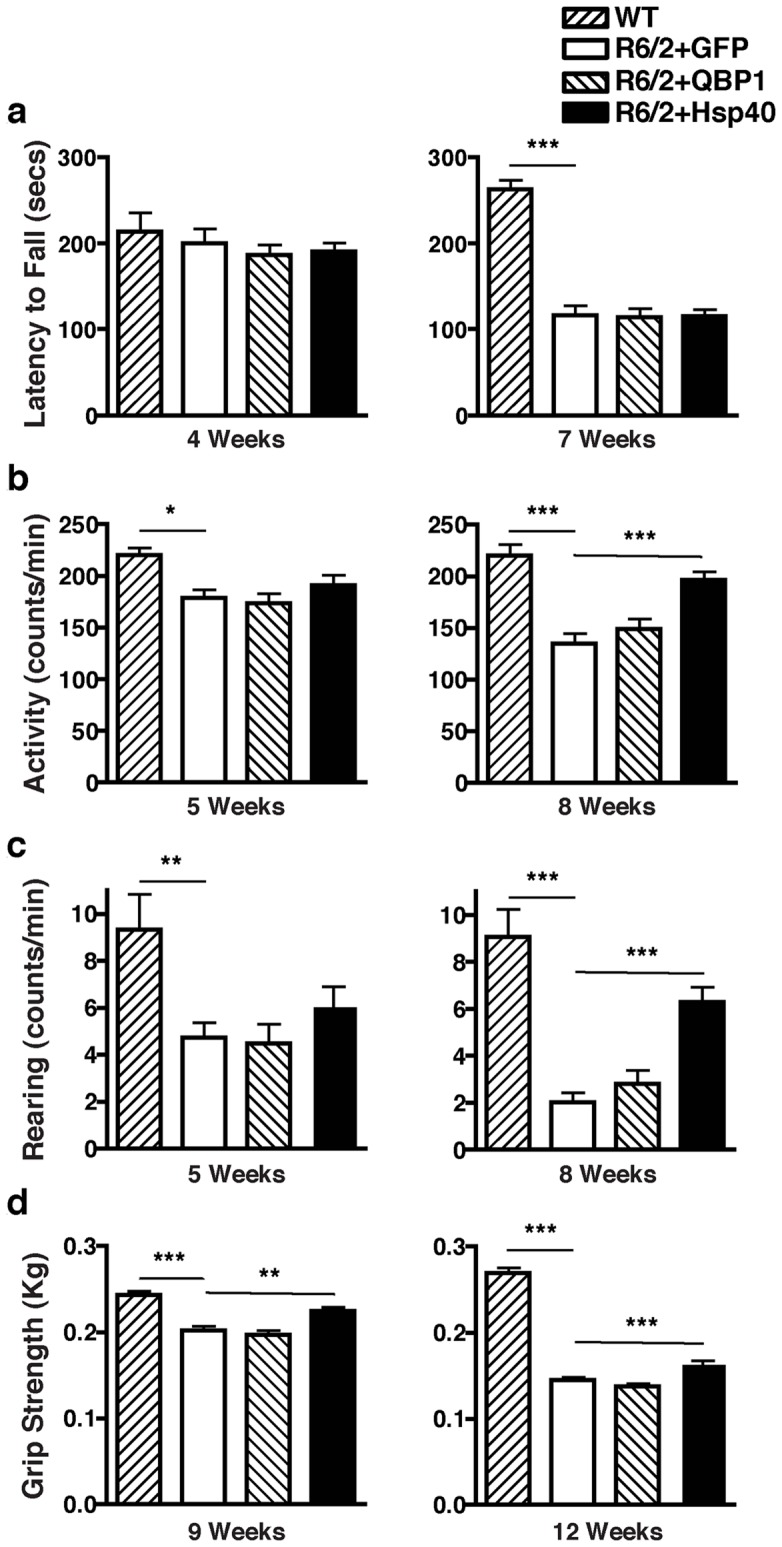
AAV5-Hsp40 improves spontaneous locomotor activity and grip strength abnormalities of polyQ disease mice. R6/2 mice at P7 were injected in both sides of the striatum with AAV5-GFP, AAV5-QBP1, or AAV5-Hsp40, and the following phenotypes were analyzed. (A) Rotarod performance measured at 4 (left) and 7 weeks (right) of age. (B, C) Open-field activity (B) and rearing (C) measured at 5 (left) and 8 weeks (right) of age. (D) Grip strength measured at 9 (left) and 12 weeks (right) of age. Values represent mean ± SEM, n≥9 mice (*p<0.05, **p<0.01, ***p<0.001).

To evaluate motor impairments of R6/2 mice, we first tested their forced locomotor activity using the rotarod. Although the reduction in rotarod performance of R6/2 mice compared with wild-type (WT) mice becomes evident by 7 weeks of age, the performance of AAV5-QBP1 injected and AAV5-Hsp40 injected R6/2 mice did not significantly differ from AAV5-GFP injected mice (Fig. 2A). We further tested the effect of these viruses on spontaneous locomotor activity. R6/2 mice demonstrated significantly less open-field activity and rearing compared with WT mice as early as 5 weeks of age, which further decreased by 8 weeks. Although we could not detect a significant improvement in AAV5-QBP1 injected mice, AAV5-Hsp40 injected R6/2 mice exhibited significantly higher open-field activity and rearing compared with AAV5-GFP injected mice at 8 weeks of age (Activity: GFP 134.8±10.2 vs Hsp40 196.4±8.4 counts/min, *p*<0.001; Rearing: GFP 2.0±0.44 vs<Hsp40 6.3±0.67 counts/min, *p*<0.001) (Figs. 2B,C). These results demonstrate the therapeutic effect of AAV5-Hsp40 on the decreased spontaneous locomotor activity of R6/2 mice.

We also tested the effect of each virus on the grip strength abnormality of R6/2 mice. Grip strength of R6/2 mice was significantly weaker than that of WT mice at 9 weeks of age, and further weakened by 12 weeks of age. AAV5-QBP1 injection did not have a significant effect on grip strength at either age. In contrast, AAV5-Hsp40 injected R6/2 mice exhibited significantly greater grip strengths than AAV5-GFP injected mice both at 9 weeks (GFP 0.202±0.005 vs Hsp40 0.225±0.005 Kg, *p*<0.01) and 12 weeks of age (GFP 0.145±0.003 vs Hsp40 0.165±0.003 Kg, *p*<0.001), demonstrating the therapeutic effect of AAV5-Hsp40 (Fig. 2D).

**Figure 3:**
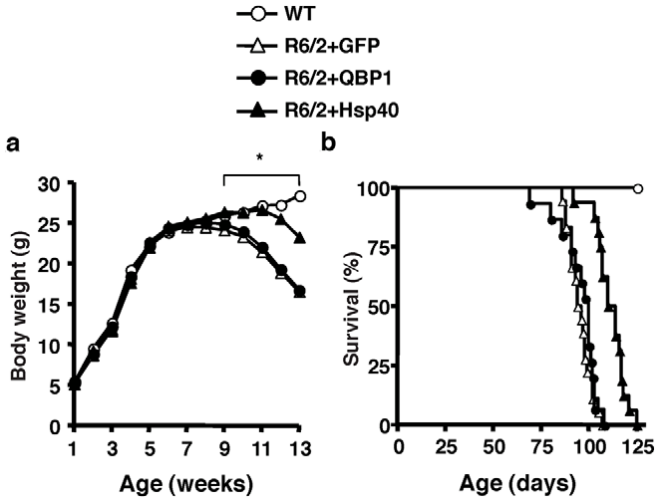
AAV5-Hsp40 improves body weight loss and extends the lifespan of polyQ disease mice. R6/2 mice at P7 were injected in both sides of the striatum with AAV5-GFP, AAV5-QBP1, or AAV5-Hsp40, and the following phenotypes were analyzed. (A) Body weight measured weekly. Values represent the mean (*p<0.05, R6/2+GFP vs R6/2+Hsp40). (B) Survival (*p*<0.0001, R6/2+GFP vs R6/2+Hsp40, Log-rank test). In both (A) and (B), n≥9 mice.

We also evaluated the body weight loss of AAV5-injected R6/2 mice. AAV5-GFP injected R6/2 mice demonstrated significantly lower body weights than WT mice after 9 weeks of age. The weights of AAV5-QBP1 injected R6/2 mice were almost indistinguishable from AAV5-GFP injected mice at each time point. In contrast, the weights of AAV5-Hsp40 injected R6/2 mice showed a significant improvement compared to AAV5-GFP injected mice between 9 and 13 weeks of age (Fig. 3A). These results demonstrate the therapeutic effect of AAV5-Hsp40 also against the weight loss of R6/2 mice.

We then assessed the effect of AAV5 injections on the lifespan of R6/2 mice. The survival of AAV5-QBP1 injected R6/2 mice (median lifespan 100 days) was not significantly different from AAV5-GFP injected mice (median lifespan 95 days). In contrast, AAV5-Hsp40 injection resulted in a rightward shift of the survival curve to a median lifespan of 112 days, demonstrating the therapeutic effect of AAV5-Hsp40 on the decreased survival of R6/2 mice (Fig. 3B). Taken together, these results clearly demonstrate that AAV5-Hsp40 significantly improves many neurological phenotypes of R6/2 mice.

### AAV5-Hsp40 Inhibits Inclusion Body Formation also in Virus Non-infected Neurons of polyQ Disease Mice

**Figure 4:**
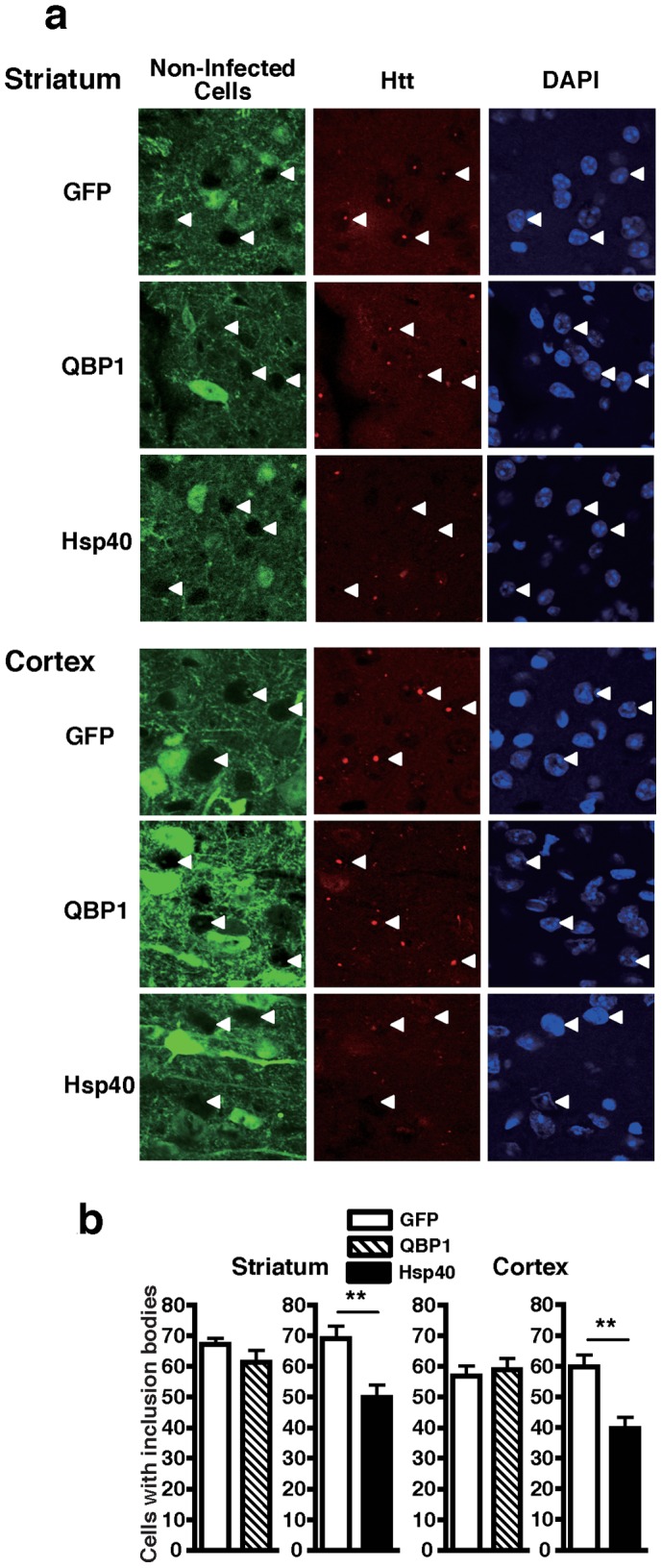
AAV5-Hsp40 also inhibits polyQ inclusion body formation in virus non-infected neurons of polyQ disease mouse brains. Htt inclusion body formation in 8 week old R6/2 mouse brains injected with AAV5-GFP on one side of the striatum and AAV5-QBP1 or AAV5-Hsp40 on the other side, was analyzed as in Fig. 1, but in the virus non-infected cells on each side of the brain. (A) Representative photographs of striatal (top panels) and cortical (bottom panels) sections of R6/2 mice injected with the indicated viruses. Green; virus infected cells, red; htt, and blue; nuclei visualized by DAPI staining. Representative virus non-infected cells in each panel are indicated by white arrowheads. (B) Inclusion body formation in virus non-infected cells on the AAV5-QBP1 injected side and AAV5-Hsp40 injected side in the striatum (left) and cortex (right). Data are shown as means ± SEM of ≥6 fields of view, in which over 180 cells were counted (**p<0.01). Representative results of two mice analyzed are shown.

When analyzing the effect of the viruses on inclusion body formation in R6/2 mice (Fig. 1), we suspected that on the AAV5-Hsp40 injected side, not only virus infected neurons, but even virus non-infected neurons appeared to have fewer inclusion bodies than those on the AAV5-GFP injected side. To clarify our suspicion, we focused on the virus non-infected neurons that are not stained with Hsp40 or GFP antibodies (see Materials and Methods and Fig. S3), and reanalyzed inclusion body formation in the virus non-infected neurons. On the AAV5-QBP1 injected side, virus non-infected neurons showed a similar rate of inclusion body formation as non-infected neurons on the AAV5-GFP injected side, in both the striatum and cortex, as expected (Fig. 4). Surprisingly, we found that on the AAV5-Hsp40 injected side, virus non-infected neurons had strikingly fewer inclusion bodies compared with non-infected neurons on the AAV5-GFP injected side in both the striatum (GFP side 69.0% vs Hsp40 side 49.8%, *p*<0.01) and cortex (GFP side 59.1% vs Hsp40 side 39.6%, *p*<0.01). The degree of inhibition was not as robust as in AAV5-Hsp40 infected neurons, but was still significant. These results raise a possibility that Hsp40 can exert a non-cell autonomous therapeutic effect on virus non-infected neurons in the brains of R6/2 mice, which is not observed with QBP1.

### Hsp40 Inhibits Secretion of Pathogenic polyQ Proteins from Cultured Cells

We next aimed to elucidate the mechanism by which Hsp40 exerts its non-cell autonomous therapeutic effect in the brains of R6/2 mice. Recent studies suggest that prion-like cell-cell transmission of aggregation-prone proteins via their release from cells and subsequent uptake into neighboring cells, is involved in the spreading of neuropathology in the polyQ diseases as well as other neurodegenerative diseases [8]–[11]. We therefore hypothesized that Hsp40 may inhibit secretion of the pathogenic polyQ protein from cells to exert its non-cell autonomous therapeutic effect.

**Figure 5:**
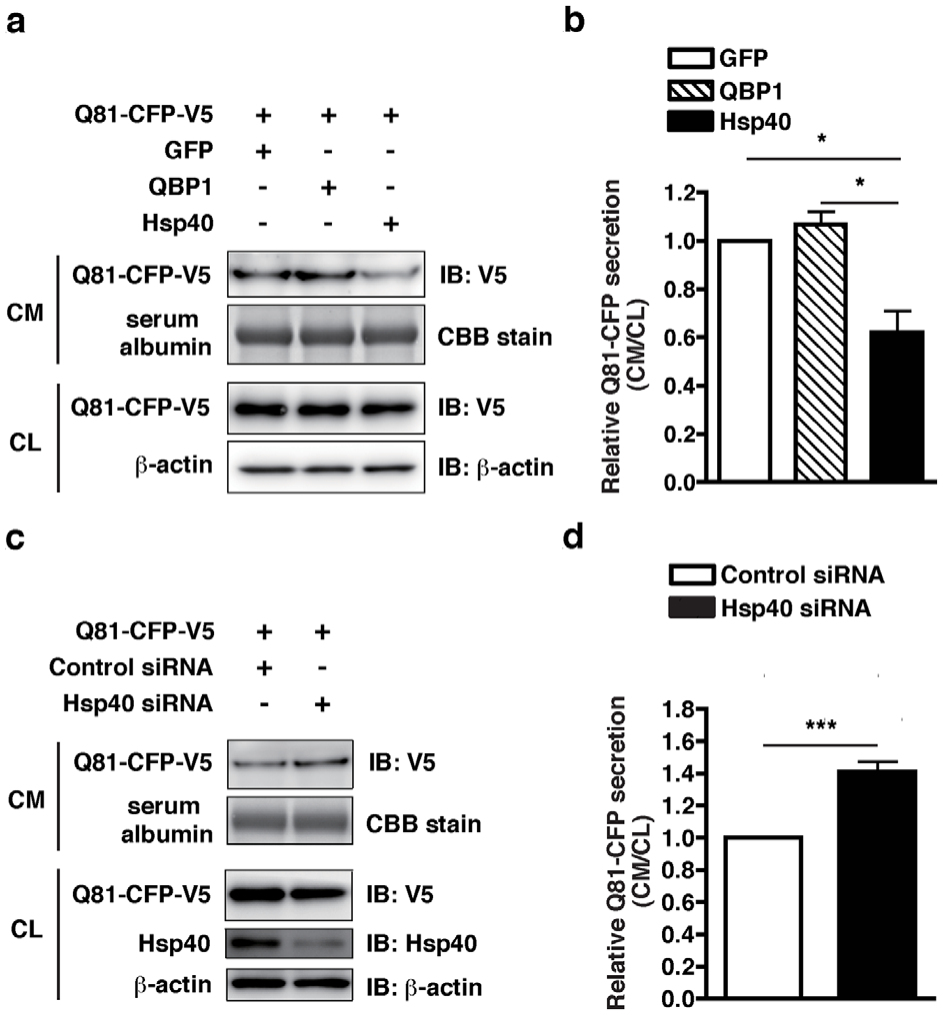
Hsp40 inhibits polyQ protein secretion in cultured cells. (A) Neuro2A cells were co-transfected with plasmids expressing Q81-CFP-V5 and either GFP, QBP1, or Hsp40, and the culture media (CM) and cell lysates (CL) were subjected to Western blot analysis with a V5 antibody to detect the Q81-CFP-V5 protein. (B) Relative amounts of Q81-CFP-V5 secreted into the culture media, calculated from the band intensities in (A), with the amount of Q81-CFP-V5 secreted from cells co-expressing GFP set to 1. (C) Neuro2A cells were transfected with a plasmid expressing Q81-CFP-V5 and siRNA against Hsp40 or a control siRNA, and the culture media (CM) and cell lysates (CL) were subjected to Western blot analysis with a V5 antibody to detect the Q81-CFP-V5 protein, and with an Hsp40 antibody. The loading controls are as in (A). (D) Relative amounts of Q81-CFP-V5 secreted into the cuture media, calculated from the band intensities in (C). In (A) and (C), serum albumin is shown as a loading control for the culture media, and β-actin as a loading control for the cell lysates. In (B) and (D), data are shown as means ± SEM of ≥ four independent experiments (*p<0.05, ***p<0.001).

We used a cell culture model to test whether Hsp40 could inhibit secretion of a pathogenic polyQ protein from cells. An expanded polyQ stretch of 81 repeats fused with CFP and a V5 tag (Q81-CFP-V5) was co-expressed together with the GFP control, QBP1, or Hsp40 in Neuro2A cells. Twenty-four h later, the culture media were replaced with fresh media to remove all of the dead cells, and after a further 6 h of incubation, culture media were collected and concentrated using centrifugal filters, and subjected to Western blot analysis. Q81-CFP-V5 was detected in the culture medium of cells (Fig. 5A), suggesting that pathogenic polyQ proteins are secreted from cells. In cells co-expressing QBP1, the amount of Q81-CFP-V5 detected in the culture medium was similar to that in cells co-expressing GFP. In contrast, Neuro2A cells co-expressing Hsp40 showed ∼40% less Q81-CFP-V5 in the culture medium compared with cells co-expressing GFP, suggesting that Hsp40 inhibits secretion of the pathogenic polyQ protein from cells (Figs. 5A,B). Furthermore, siRNA-mediated knockdown of endogenous Hsp40 increased the secretion of Q81-CFP-V5 by ∼40% compared with cells treated with a control siRNA (Figs. 5C,D), indeed confirming that Hsp40 inhibits polyQ protein secretion. These results imply that inhibition of secretion of the polyQ protein from cells by Hsp40 results in a non cell-autonomous therapeutic effect in R6/2 mouse brains, possibly via inhibition of the cell-cell transmission of the pathogenic polyQ protein.

## Discussion

In this study we show for the first time that viral vector-mediated expression of a molecular chaperone, namely Hsp40 significantly improves the neurological phenotypes of a mouse model of the polyQ diseases. Although a recent study reported the effectiveness of another Hsp40 family member, HSJ1a (DNAJB2a) in R6/2 mice, this study cannot be directly translated to a therapy since it was performed by the crossing of transgenic mice [30]. In addition, although lentiviral vector-mediated delivery of DNAJB2a to a polyQ disease rat model has been investigated [37], it did not demonstrate the therapeutic effect on the neurological phenotypes, perhaps because of the limited spread of lentiviruses. We successfully overcame these above problems by using AAV, which infects a widespread area of the brain, and is already used in human patients [38].

We did not detect significant therapeutic effects of AAV5-Hsp70 on R6/2 mice unlike AAV5-Hsp40, possibly due to the very low infection rate of our AAV5-Hsp70 (data not shown), or differences in the effectiveness of Hsp40 and Hsp70 against mutant htt. Indeed, previous studies examining the effect of Hsp70 in R6/2 mice have shown no or very modest therapeutic effects [28], [29]. Furthermore, a cell culture study demonstrated that Hsp40 family members are effective against the toxicity of mutant htt, while Hsp70 family members are ineffective [35]. Taken together, these studies indicate that Hsp40 family members may be more effective than Hsp70 family members against the toxic effects of mutant htt.

We surprisingly found that AAV5-Hsp40 inhibits inclusion body formation also in virus non-infected cells (Fig. 4), suggesting a non cell-autonomous therapeutic effect. Aggregation prone proteins that cause neurodegenerative diseases including pathogenic polyQ proteins, as well as α-synuclein which causes Parkinson’s disease, and tau which causes the tauopathies have recently been suggested to be transmitted between cells, and this may be the mechanism leading to the progressive spread of neuropathology in these diseases [8]–[11]. We detected a significant amount of the pathogenic polyQ protein in the culture medium of Neuro2A cells (Fig. 5A), suggesting its cell-cell transmission, which is compatible with previous studies [39], [40]. We further found that Hsp40 suppresses secretion of the pathogenic polyQ protein from cells (Fig. 5), suggesting that it may inhibit the cell-cell transmission of the pathogenic polyQ protein. The non-cell autonomous therapeutic effect of Hsp40 may also involve other mechanisms, such as (1) AAV5-Hsp40 infected neurons may create a better environment for contacting non-infected neurons [41] or (2) Hsp40 itself may be secreted to exert therapeutic effects on neighboring non-infected neurons, as is suggested for Hsp70 [42].

We were unable to detect the therapeutic effect of AAV5-QBP1 on the neurological phenotypes of R6/2 mice, although we successfully detected its inhibition of inclusion body formation. However, we and others have previously shown that expression of QBP1 exerts therapeutic effects on the neurological phenotypes of *Drosophila* and mouse models of the polyQ diseases [16], [17], [43]. Since the infection efficiency of all of the viruses used in this study was quite low (∼30%), the extent of neurons expressing QBP1 in the R6/2 mouse brains was probably insufficient to exert a detectable effect on the phenotypes. On the other hand, in the case of AAV5-Hsp40, inhibition of polyQ protein secretion which should lead to an increase in the number of rescued neurons, likely contributed to its improvement of the neurological phenotypes. Other possibilities may also contribute to their varying effects, for example (1) Hsp40 is more effective than QBP1 in inhibiting polyQ protein misfolding/aggregation, and (2) Hsp40 can also support the degradation of misfolded proteins [19], while QBP1 cannot.

In this study we demonstrate a therapeutic strategy against the polyQ diseases using AAV5-Hsp40, which has great potential for clinical application, since AAVs are safe and are widely utilized in clinical trials [38]. We further suggest a novel therapeutic mode of action of Hsp40, namely suppression of pathogenic polyQ protein secretion from cells, which may consequently suppress its cell-cell transmission. Since the transmission of aggregation-prone proteins is thought to be involved also in other neurodegenerative diseases, Hsp40 may exert a non-cell autonomous therapeutic effect on these other diseases. Elucidation of how Hsp40 inhibits polyQ protein secretion should reveal new therapeutic targets and strategies for neurodegenerative diseases caused by aggregation-prone proteins.

## Materials and Methods

### Viral Vectors

Adeno-associated virus type 5 (AAV5) vector plasmids contained an expression cassette with a human cytomegalovirus enhancer/chicken β actin (CAG) promoter followed by the first intron of human growth hormone, target cDNA (either a tandem repeat of QBP1 fused to GFP [14], human Hsp40 (DNAJB1) [44], or GFP), and a simian virus 40 polyadenylation signal sequence, all positioned between the inverted terminal repeats of the AAV5 genome. AAV5 vectors were produced using the AAV5 plasmid, the AAV5 helper plasmid containing the rep and cap sequences from AAV5, as well as the pHelper plasmid from the AAV Helper-Free System containing the E2A, E4, and VA RNA genes of the adenovirus genome (Stratagene, La Jolla, CA). HEK293 cells were co-transfected with the AAV5 plasmid and two helper plasmids by the calcium phosphate method. Seventy-two h later, the cells were harvested and subjected to three rounds of freeze-thaw lysis. AAV5 vectors were then purified by two rounds of cesium chloride density gradient centrifugation. Vector titers were estimated by quantitative DNA dot-blot hybridization to be ∼0.2–1.6×10^13^ genome copies/ml.

### Animals

All animal experiments were performed in accordance with the guidelines of the Animal Ethics Committee of the National Institute of Neuroscience, National Center of Neurology and Psychiatry, Japan, and performed in accordance with the guidelines. Mice transgenic for human *huntingtin* exon 1 with approximately 150 CAG repeats (strain R6/2) [33] were obtained from the Jackson Laboratory (Bar Harbor, ME), and maintained on a B6CBAF1 background. Genotypes were analyzed and CAG repeat numbers of the transgenic mice were confirmed to be similar by PCR as previously described [33]. Mice were housed on a 12-hour light/dark cycle, with food and water provided *ad libitum*. At least nine male R6/2 mice per group and wild-type littermate (WT) controls were used for the phenotype analyses, and two R6/2 mice were used for the inclusion body analyses.

### AAV Injections

P7 old R6/2 mice were stereotaxically injected with 1 µl of virus solution (AAV5-GFP, AAV5-QBP1, or AAV5-Hsp40) into the striatum (coordinates 1 mm anterior to bregma, 2.25 mm lateral to the midline, and 3 mm ventral to the skull surface) at a rate of 0.1 µl/min using a 10 µl Hamilton syringe (Hamilton Company, Reno, NV) and an infusion pump (KD Scientific, Holliston, MA). For inclusion body analyses, AAV5-GFP was injected into one side of the striatum and AAV5-QBP1 or AAV5-Hsp40 into the other side. For phenotype analyses, the same virus was injected into both sides of the striatum.

### Mouse Phenotype Analyses

For rotarod analysis, mice were tested at 4 and 7 weeks of age on an accelerating rotarod apparatus (Ugo Basile, Comerio, Italy) set to accelerate from 4 to 40 rpm over a period of 300 seconds. The time it took for each mouse to either fall off the rod or cling onto the rod for one full rotation was recorded. Mice were tested on the rod for three consecutive days, with three trials per day. The first day was regarded as training, and only the data from the second and third day were used. The highest and lowest values were excluded, and the middle four values were averaged. Grip strength analysis was performed at 9 and 12 weeks of age using a grip strength meter (Muromachi Kikai, Tokyo, Japan). Mice were placed gently by their tail on the metal grid of the meter so that they grip the grid with both forelimbs and hindlimbs, at which point they were pulled back gently with their tails, exerting a tension that is measured by the meter. Five trials were performed for each mouse, and the highest and lowest values were excluded and the middle three values were averaged. To measure open-field activity and rearing, mice at 5 and 8 weeks of age were tested using a spontaneous locomotor activity monitor (Supermex, Muromachi Kikai, Tokyo, Japan), consisting of an acrylic box of dimensions 40 cm×28 cm×31 cm with an activity sensor placed at the top and rearing sensors placed at the sides of the box. Mice were left in the box for a total of 15 min, and their activity count and rearing frequency were measured. For body weight analysis mice were weighed weekly. Mice were followed until their deaths in order to calculate their lifespans, a widely accepted and valuable parameter to assess therapeutic effects in these mice, which was approved by the institution’s Animal Ethics Committee.

### Tissue Preparation and Immunohistochemical Analyses

Mice were deeply anesthetized with sodium pentobarbital (100 mg/kg), and then perfused intercardially with saline followed by 4% paraformaldehyde (PFA) in phosphate-buffered saline (PBS). Brains were removed, post-fixed in 4% PFA in PBS at 4°C overnight, and then cryoprotected in 30% sucrose in PBS at 4°C for 24 h. Frozen 10 µm sections were cut using a cryostat. For immunohistochemical analysis, sections were blocked in PBS containing 5% goat serum and 0.1% Triton X-100 for 1 h at room temperature. The sections were then incubated with a mouse anti-huntingtin antibody (1∶250; MAB5374, Millipore, Billerica, MA), and either a rabbit anti-Hsp40 antibody (1∶500; SPA-400, Enzo Life Sciences, Farmingdale, NY) or a rabbit anti-GFP antibody (1∶250; A11122, Invitrogen, Carlsbad, CA) at 4°C overnight, followed by an Alexa Fluor 596-conjugated goat anti-mouse IgG antibody and an Alexa Fluor 488 goat anti-rabbit IgG antibody (1∶1000, Invitrogen) for 1 h at room temperature. Sections were mounted with Slowfade Gold antifade reagent with DAPI (Invitrogen) and examined using a confocal laser scanning microscope (FV1000; Olympus, Tokyo, Japan). The average fluorescence intensity of representative cells that were regarded as either “infected” or “non-infected” in each sample were measured using the Image J software, confirming that the two population of cells can readily be distinguished in the images (Fig. S3).

### Cell Culture, Transfection and Western Blot Analysis

Neuro2A cells (obtained from ATCC) were grown and maintained in DMEM supplemented with 10% (v/v) FBS. Cells were plated on a 35-mm dish at a density of 4×10^5^ cells per dish on the day before transfection. For the overexpression experiment, a plasmid vector encoding a tandem repeat of QBP1 fused to GFP, human Hsp40 [44], or GFP was transiently co-transfected with the Q81-CFP-V5 vector encoding an expanded polyQ stretch of 81 repeats fused with CFP and a V5 tag, using Lipofectamine LTX with PLUS reagent (Invitrogen). For the knockdown experiment, siRNA targeted against mouse Hsp40 (Santa Cruz Biotechnology, Santa Cruz, CA) or a control siRNA (RNAi Inc, Tokyo, Japan) was cotransfected with the Q81-CFP-V5 vector using Lipofectamine 2000 reagent (Invitrogen). At 24 h after transfection, culture media were replaced with fresh media, and after a further 6 h of incubation, culture media were collected and whole cell lysates were prepared with 1% Triton X in tris-buffered saline. For Western blot analysis, the culture media were concentrated using 30 kDa cutoff Amicon Ultra filters (Millipore). Q81-CFP-V5 in the concentrated culture media and whole cell lysates was separated using 10% SDS-PAGE gels and transferred onto PVDF membranes (Bio-Rad, Hercules, CA). The membranes were incubated overnight with an HRP-conjugated anti-V5 antibody (1∶2500, Invitrogen) at 4°C. The HRP signal was visualized with SuperSignal West Femto Chemiluminescent Substrate (Thermo Fisher Scientific Inc, Rockford, IL), captured with a LAS-3000 mini CCD imaging system (Fujifilm, Tokyo, Japan), and band intensities were quantified using the Image J software. For the siRNA experiments, the membranes were then stripped and incubated with a rabbit anti-Hsp40 antibody (1∶5000, SPA-400, Enzo Life Sciences Inc, Farmingdale, NY) at 4°C followed by an HRP-conjugated anti-rabbit IgG secondary antibody (1∶10000, Thermo Fisher Scientific), and visualized as above.

### Statistical Analyses

For the rotarod, open-field activity, rearing, grip strength and weight data, statistical analyses were performed by using one-way analysis of variance followed by Tukey’s multiple comparison test to assess for significant differences between individual groups. The survival data was analyzed using the Log-rank test. For the inclusion body formation and Western blot analyses, Student’s *t*-test was used. For all analyses, *p*<0.05 was considered as significant.

## Supporting Information

Figure S1 **AAV5 injection into the mouse striatum at P7 results in widespread expression of the transgene.** R6/2 mice at P7 were injected in the right striatum with 1 µl of AAV5-QBP1, and 2 weeks later the expression of QBP1 was analyzed by immunohistochemistry. This widespread expression of QBP1 throughout the brain lasts for at least 13 weeks (data not shown).
(PDF)

Figure S2 **AAV5-QBP1 inhibits polyQ inclusion body formation in virus infected neurons of polyQ disease mice.** R6/2 mice at P7 were injected with AAV5-GFP on one side of the striatum and AAV5-QBP1 on the other side, and at 4, 8, and 14 weeks of age htt inclusion body formation in virus infected neurons of the striatum (left) and cortex (right) was assessed by immunohistochemistry. Data are shown as means ± SEM of ≥ 6 fields of view, in which over 180 cells were counted (*p<0.05, ***p<0.001). Representative results of two mice analyzed are shown.
(PDF)

Figure S3 **AAV5 “infected” and “non-infected” cells can be clearly distinguished from their fluorescence intensity.** The fluorescence intensity of representative cells that were regarded as either “infected” or “non-infected” in photographs of immunostained brain sections of R6/2 mice injected with either AAV5-GFP (left), AAV5-QBP1 (middle) or AAV5-Hsp40 (right). For each sample, a total of over 100 representative cells were analyzed from 5–6 fields of view.
(PDF)
