# Supplementary figures and images for: Hsp40 Gene Therapy Exerts Therapeutic Effects on Polyglutamine Disease Mice via a Non-Cell Autonomous Mechanism
Source: PLoS One. 2012 Nov 30;7(11):e51069. doi: 10.1371/journal.pone.0051069 (PMC3511362; doi:10.1371/journal.pone.0051069)

**Supplementary Fig. S1**

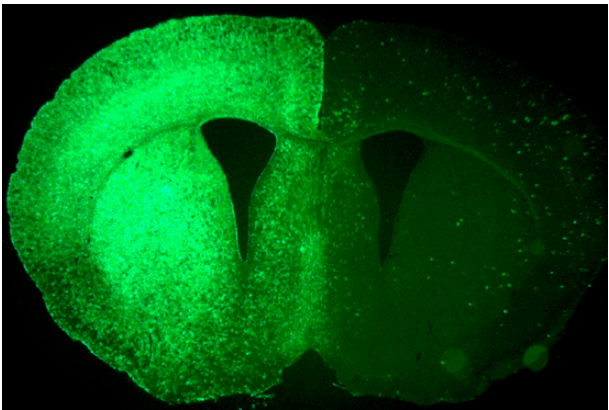

Supplement: Figure S1 — AAV5 injection into the mouse striatum at P7 results in widespread expression of the transgene. R6/2 mice at P7 were injected in the right striatum with 1 µl of AAV5-QBP1, and 2 weeks later the expression of QBP1 was analyzed by immunohistochemistry. This widespread expression of QBP1 throughout the brain lasts for at least 13 weeks (data not shown). (PDF) [file pone.0051069.s001.pdf]

## Supplementary Fig. S2

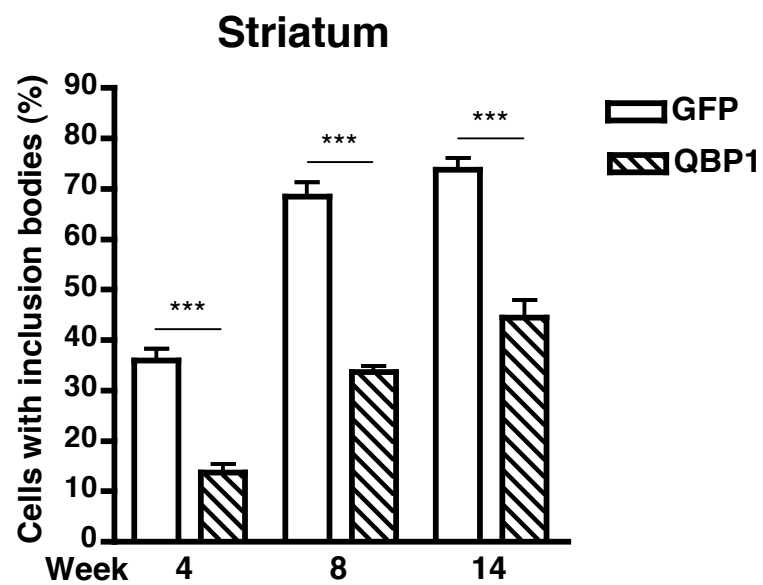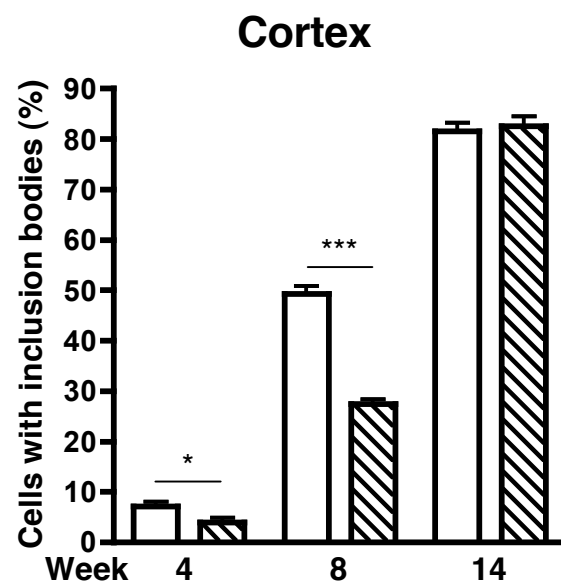

Supplement: Figure S2 — AAV5-QBP1 inhibits polyQ inclusion body formation in virus infected neurons of polyQ disease mice. R6/2 mice at P7 were injected with AAV5-GFP on one side of the striatum and AAV5-QBP1 on the other side, and at 4, 8, and 14 weeks of age htt inclusion body formation in virus infected neurons of the striatum (left) and cortex (right) was assessed by immunohistochemistry. Data are shown as means ± SEM of ≥ 6 fields of view, in which over 180 cells were counted (*p<0.05, ***p<0.001). Representative results of two mice analyzed are shown. (PDF) [file pone.0051069.s002.pdf]

# Supplementary Fig. S3

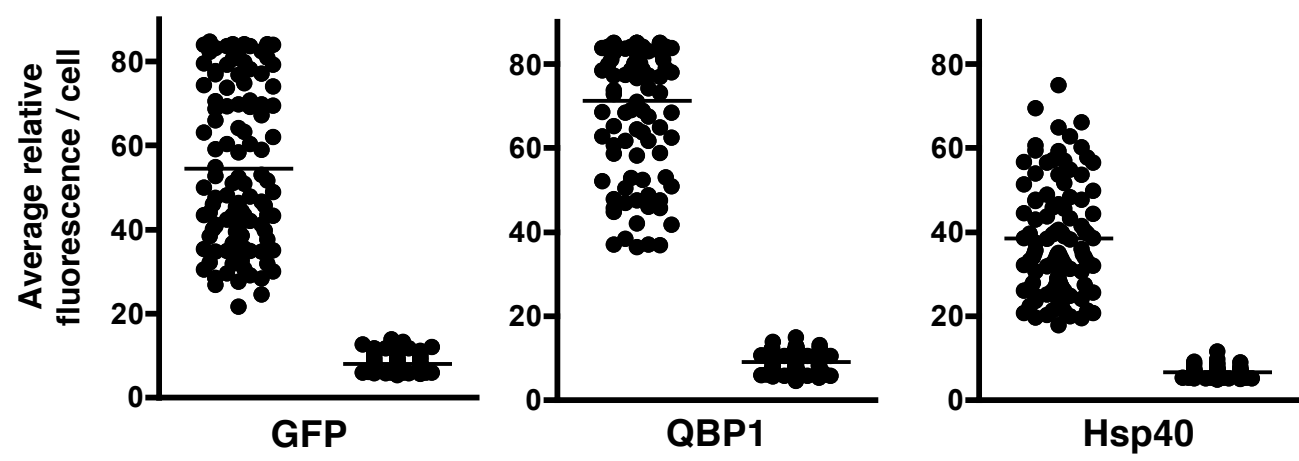

Supplement: Figure S3 — AAV5 “infected” and “non-infected” cells can be clearly distinguished from their fluorescence intensity. The fluorescence intensity of representative cells that were regarded as either “infected” or “non-infected” in photographs of immunostained brain sections of R6/2 mice injected with either AAV5-GFP (left), AAV5-QBP1 (middle) or AAV5-Hsp40 (right). For each sample, a total of over 100 representative cells were analyzed from 5–6 fields of view. (PDF) [file pone.0051069.s003.pdf]
